# Retinoic acid attenuates ischemic injury-induced activation of glial cells and inflammatory factors in a rat stroke model

**DOI:** 10.1371/journal.pone.0300072

**Published:** 2024-03-25

**Authors:** Ju-Bin Kang, Hyun-Kyoung Son, Murad-Ali Shah, Phil-Ok Koh

**Affiliations:** Department of Anatomy and Histology, College of Veterinary Medicine, Research Institute of Life Science, Gyeongsang National University, Jinju, South Korea; Massachusetts General Hospital/Harvard Medical School, UNITED STATES

## Abstract

Stroke is a leading cause of death and long-term disability which can cause oxidative damage and inflammation of the neuronal cells. Retinoic acid is an active metabolite of vitamin A that has various beneficial effects including antioxidant and anti-inflammatory effects. In this study, we investigated whether retinoic acid modulates oxidative stress and inflammatory factors in a stroke animal model. A middle cerebral artery occlusion (MCAO) was performed on adult male rats to induce focal cerebral ischemia. Retinoic acid (5 mg/kg) or vehicle was injected into the peritoneal cavity for four days before MCAO surgery. The neurobehavioral tests were carried out 24 h after MCAO and cerebral cortex tissues were collected. The cortical damage was assessed by hematoxylin-eosin staining and reactive oxygen species assay. In addition, Western blot and immunohistochemical staining were performed to investigate the activation of glial cells and inflammatory cytokines in MCAO animals. Ionized calcium-binding adapter molecule-1 (Iba-1) and glial fibrillary acidic protein (GFAP) were used as markers of microglial and astrocyte activation, respectively. Tumor necrosis factor-α (TNF-α) and interleukin-1β (IL-1β) were used as representative pro-inflammatory cytokines. Results showed that MCAO damage caused neurobehavioral defects and histopathological changes in the ischemic region and increased oxidative stress. Retinoic acid treatment reduced these changes caused by MCAO damage. We detected increases in Iba-1 and GFAP in MCAO animals treated with vehicle. However, retinoic acid alleviated increases in Iba-1 and GFAP caused by MCAO damage. Moreover, MCAO increased levels of nuclear factor-κB and pro-inflammatory cytokines, including TNF-α and IL-1β. Retinoic acid alleviated the expression of these inflammatory proteins. These findings elucidate that retinoic acid regulates microglia and astrocyte activation and modulates pro-inflammatory cytokines. Therefore, this study suggests that retinoic acid exhibits strong antioxidant and anti-inflammatory properties by reducing oxidative stress, inhibiting neuroglia cell activation, and preventing the increase of pro-inflammatory cytokines in a cerebral ischemia.

## Introduction

Stroke is a leading cause of long-term disability and death [1]. It is divided into two types: ischemic stroke and hemorrhagic stroke. Ischemic stroke due to vascular occlusion is the most common type of stroke. A hemorrhagic stroke is caused by a burst blood vessel. Ischemic stroke occurs when blood flow in the cerebral artery is temporarily or permanently blocked [2, 3]. Ischemic conditions caused by blockages of blood vessels decrease the blood supply to brain tissue, cause oxygen and glucose deprivation and eventually leads to cerebral infarction [2]. Moreover, ischemia produces excitotoxicity and oxidative damage, induces blood-brain barrier dysfunction, and causes an inflammatory response [3].

Inflammation is an important defense mechanism of the body in response to a variety of harmful stimuli, including pathogens, radiation, and toxicants [4–6]. The inflammatory response of the brain is activated by nerve excitability, nerve damage, and blood-brain barrier permeability. Thus, brain inflammation is considered to be the cause of neurological diseases such as Parkinson’s disease, traumatic brain injury, and ischemic stroke [1, 7, 8]. Ischemic injury activates glial cells such as microglia and astrocytes, activates pro-inflammatory cytokines including tumor necrosis factor-α (TNF-α) and interleukin-1β (IL-1β), damages the blood-brain barrier, and results in neuronal damage [9, 10]. Moreover, activation of glial cells is involved in an inflammatory response mediated by nuclear factor-kappa B (NF-κB) [11]. NF-κB is a protein complex that regulates various biological functions, including inflammation, immune responses, and cell survival [12, 13]. Activation of NF-κB plays an important role in cerebral ischemic stroke by regulating inflammation and oxidative stress [14]. Ischemic stroke induces activation of NF-κB, overproduction of inflammatory factors such as TNF-α and IL-1β, activation of caspase proteins, and induction of neuronal cell death [15].

Retinoic acid is an active metabolite of vitamin A that is required for development of many organs, including the eyes, spinal cord, and brain [16]. This substance has antioxidant, anti-inflammatory, and anti-apoptotic properties [17–19]. It is also involved in cell proliferation, differentiation, and apoptosis [20–23]. In addition, retinoic acid exerts neuroprotective effects on the brain by preventing blood-brain barrier dysfunction, relieving microglia and astrocyte activation, and reducing the release of inflammatory cytokines [22, 23]. We previously demonstrated the neuroprotective effect of retinoic acid by modulating apoptosis-associated proteins in cerebral ischemia [24]. Although the neuroprotective effects of retinoic acid in ischemic injury have been reported, the exact mechanisms of retinoic acid in cerebral ischemia remain ambiguous and complex. Therefore, studies on the neuroprotective mechanisms of retinoic acid are needed. This study focused on the regulation of inflammatory responses by retinoic acid in an ischemic stroke model. The purpose of this study was to investigate the antioxidant and anti-inflammatory effects of retinoic acid and to determine whether retinoic acid regulates the expression of inflammatory proteins, such as NF-κB, TNF-α, and IL-1β.

## Materials and methods

### Experimental animals and drug treatment

Male Sprague Dawley rats (210–220 g, *n =* 40) were provided by the Samtako Co. (Animal Breeding Centre, Osan, Korea). Animals were moved to cages and allowed to rest for a week in controlled environmental conditions (25ºC, 12 h light/12 h) to adapt to the new environment. They were divided into four groups: vehicle + sham, retinoic acid + sham, vehicle + middle cerebral artery occlusion (MCAO), and retinoic acid + MCAO groups. Retinoic acid (5 mg/kg, Sigma Aldrich, St. Louis, MO, USA) was dissolved in a dissolving solution (polyethylene glycol, 0.9% Nacl, and ethanol; 70%/20%/10% by volume) and injected into the abdominal cavity for four days prior to MCAO surgery. The dose and treatment time of retinoic acid were determined by a previously reported method [25]. Animals in the vehicle group were injected with only a dissolving solution. All animals were used for neurobehavioral tests and brain tissue was collected for further experiments. Five animals from each group were used for histological analysis including hematoxylin and eosin staining and immunofluorescence staining. Five animals from each group were used for oxidative stress and Western blot analyses. All experiments were performed by following the guidelines of the Institutional Animal Care and Use Committee of Gyeongsang National University (GNU-210302-R0023).

### Middle cerebral artery occlusion

MCAO surgery was performed according to a previously described method [26]. Animals were anesthetized by intramuscular injection using Zoletil (50 mg/kg, Virbac, Carros, France). They were placed in a dorsal recumbency on a heating pad to prevent hypothermia and the right side of the neck was opened with a midline incision. The right common carotid artery (CCA) was separated from surrounding tissues and nerves, and the right external carotid artery (ECA) and right internal carotid artery (ICA) were continuously exposed. The right CCA was temporarily blocked with a microvascular clamp and the proximal end of right ECA was cut. A nylon suture (4/0) with a rounded tip made by flame was inserted into the right ECA. The nylon suture was moved to the middle cerebral artery (MCA) through the ICA until resistance was felt. The nylon suture was ligated with the right ECA and the neck was closed with a black suture. MCAO was maintained for 24 h and animals were sacrificed by cervical dislocation. Whole brain tissues were collected for histological staining, and right cerebral cortex tissues were collected for oxidative stress and protein level analysis and stored at -70ºC until next experiment.

### Corner test

Corner test was performed to assess sensorimotor and postural dysfunction according to a previously described procedure [27]. Two white boards (30 × 20 × 1 cm^3^) were placed at an angle of 30° to make a corner with a small space. Animals were allowed to enter and move into the corner. When both sides of vibrissae were stimulated, animals turned left or right to exit the corner. Animals were trained for 7 days prior to MCAO surgery and the corner test was performed 24 h after MCAO surgery. Corner test was repeated ten times and the number of left or right turns was recorded.

### Grip strength test

The left and right forelimb grip strength was measured as previously described [28] using the grip strength meter (Jeung Do Bio & Plant Co., Ltd., Seoul, Korea). Animals were pulled by the tail and the maximum force was measured. One forelimb was wrapped with an adhesive tape to measure the grip strength of the other forelimb and the test was performed five times on each forelimb.

### Hematoxylin and eosin staining

The whole brains were dissected 24 h after MCAO surgery and cut into 5 mm thickness. The brain tissues were fixed in 4% neutral buffered paraformaldehyde solution and washed overnight with tap water. They were dehydrated with graded ethyl alcohol (70% to 100%), cleaned with xylene, and embedded in paraffin using a paraffin embedding center (Leica, Wetzlar, Germany). The paraffin blocks were cut into 4 μm thickness using a rotary microtome (Leica), mounted on slide glasses, and dried in a slide warmer (Thermo Fischer Scientific, Waltham, MA, USA). The slides were deparaffinized in xylene, rehydrated with graded ethyl alcohol (100%-70%), and transferred to tap water. They were stained in Harris’ hematoxylin solution (Sigma-Aldrich) and washed with running tap water. They were differentiated in 1% hydrochloric acid solution with 70% ethyl alcohol, dipped in tap water, neutralized in 1% ammonia solution, and immersed in tap water. The slides were stained with eosin Y solution (Sigma-Aldrich), dehydrated with graded ethyl alcohol (70% to 100%), and rinsed with xylene. They were coverslipped with permount mounting medium (Thermo Fischer Scientific) and the right cerebral cortical region was observed and photographed with an Olympus microscope (Olympus, Tokyo, Japan).

### Reactive oxygen species assay

Reactive oxygen species (ROS) levels were detected 24 h after MCAO using a ROS assay kit (Elabscience Biotechnology Inc. Houston, TX, USA). We followed the single suspension method according to the manual provided by the manufacturer. The right cerebral cortex tissue (10 mg) was dissolved, treated with a cooled reagent working solution, and incubated with an enzyme solution at 37°C for 30 min to digest the cortical tissue. The digestion was stopped with a reagent 3 working solution, centrifuged at 500 g for 10 min, and the supernatant was discarded. They were washed with reagent 3 working solution, centrifuged at 500 g for 10 min, and mixed with reagent 1 working solution. The mixture was incubated with 5 mM 2’, 7’-dichlorodihydrofluorescein diacetate at 37°C for 1 h to produce 2’, 7’-dichlorodihydrofluorescein (DCF). They were centrifuged at 1,000 g for 10 min and the cells were collected. The remaining cells were washed with a reagent 3 working solution and fluorescence values were observed at an excitation wavelength of 500 nm and an emission wavelength of 525 nm using a spectrophotometer. The result was expressed as the ratio of the other groups to the intensity of vehicle + sham group. The intensity of vehicle + sham group was set to 1.

### Lipid peroxidation assay

Lipid peroxidation (LPO) assay was performed to evaluate the levels of oxidative stress. We measured the level of malondialdehyde (MDA), a marker of oxidative stress, by LPO assay kit (Biovision Incorporated, Milpitas, CA, USA) according to the guidelines. The tissue of the right cerebral cortex (10 mg) was dissolved in an MDA lysis buffer containing butylated hydroxytoluene and centrifuged at 13,000 g for 10 min. The supernatant was collected, mixed with thiobarbituric acid, incubated at 95°C for 1 h, and cooled in ice. The absorption value was observed at a wavelength of 532 nm using a spectrophotometer, and the result was expressed as nmol/mg of protein.

### Western blot analysis

Western blot analysis was conducted according to the reported manual [29]. The right cerebral cortex tissues (100 mg) were dissected 24 h after MCAO and kept at -70ºC. They were liquefied in a lysis buffer [1% Triton X-100, 1 mM EDTA in 1 × phosphate buffered saline (PBS, pH 7.4)] containing 200 μM phenylmethylsulfonyl fluoride. The homogenized tissues were sonicated for 3 min, centrifuged at 15,000 g for 20 min, and the supernatant was collected. Protein concentrations of each sample were measured using a bicinchoninic acid protein assay kit (Pierce, Rockford, IL, USA). Proteins (30 μg) from each group were loaded onto 10% sodium dodecyl sulfate-polyacrylamide gel and electrophoresed at 10 mA for 30 min and at 20 mA until the dye reached the bottom. They were transferred to a polyvinylidene difluoride membranes at 120 V for 2 h. The membrane was incubated with a 5% skim milk solution for 1 h to block non-specific antibody binding. They were rinsed with a tris-buffered saline solution with 0.1% Tween 20 (TBST) and incubated at 4ºC overnight with the following primary antibodies: anti-ionized calcium binding adaptor molecule-1 (Iba-1), anti-glial fibrillary acidic protein (GFAP), anti-NF-κB (diluted 1:1,000, Cell Signaling Technology, Danvers, MA, USA), anti-TNF-α, anti-IL-1β, and anti-β-actin (diluted 1:1,000, Santa Cruz Biotechnology, Dallas, TX, USA). The membranes were washed with TBST, reacted with secondary antibodies (anti-mouse IgG or anti-rabbit IgG, dilution 1:5,000, Cell Signaling Technology) for 2 h at room temperature, and then washed again with TBST. They were treated with chemiluminescent detection reagents (GE Healthcare, Little Chalfont, Buckinghamshire, UK) to detect bands on X-ray films (Fuji Film, Tokyo, Japan). X-ray films were placed on the membrane, developed in the developer solution, fixed in the fixation solution, and dried. They were scanned with a scanner and the band intensities was analyzed by Image J (Media Cybernetics, Rockville, MD, USA). The relative density of proteins was expressed by the density of each protein relative to the density of β-actin.

### Immunofluorescence staining

Immunofluorescence staining was performed using the previously reported method [30].The paraffin sections were deparaffinized with xylene and rehydrated with graded ethyl alcohol (100% to 70%). They were washed with PBS and reacted with proteinase K (Thermo Fischer Scientific). They were incubated with 1% normal goat serum for 1 h at room temperature to prevent non-specific antibodies bindings, and incubated with the following primary antibodies at 4°C overnight: anti-Iba-1, anti-GFAP, anti-NF-κB (diluted 1:100, Cell Signaling Technology), anti-TNF-α, and anti-IL-1β (diluted 1:100, Santa Cruz Biotechnology). The sections were washed with PBS, treated with fluorescein isothiocyanate-conjugated secondary antibody or tetramethylrhodamine-isothiocyanate conjugated secondary antibody (diluted 1:100, Santa Cruz Biotechnology) for 90 min at room temperature, and washed with PBS. They were counterstained with 4′,6-diamidino-2-phenylindole (DAPI, Sigma-Aldrich) and coverslipped with fluorescent mounting medium (Dako, North America Inc., USA). The stained slides were observed and images of the right cerebral cortex were obtained using a confocal microscope (FV-1000, Olympus). The integrated densities were analyzed with Image J. They were expressed as a ratio of the density of each animals to that of vehicle + sham animals.

### Statistical analysis

All experimental data in this study is shown as a mean ± standard error of mean (S.E.M.). Two-way analysis of variance (ANOVA) and the post-hoc Scheffe’s test were performed to analyze the variations among groups. p<0.05 was considered to be statistically significant.

## Results

### Retinoic acid alleviates neurobehavioral deficits, histopathological changes, and oxidative stress production in cerebral ischemia

**Fig 1:**
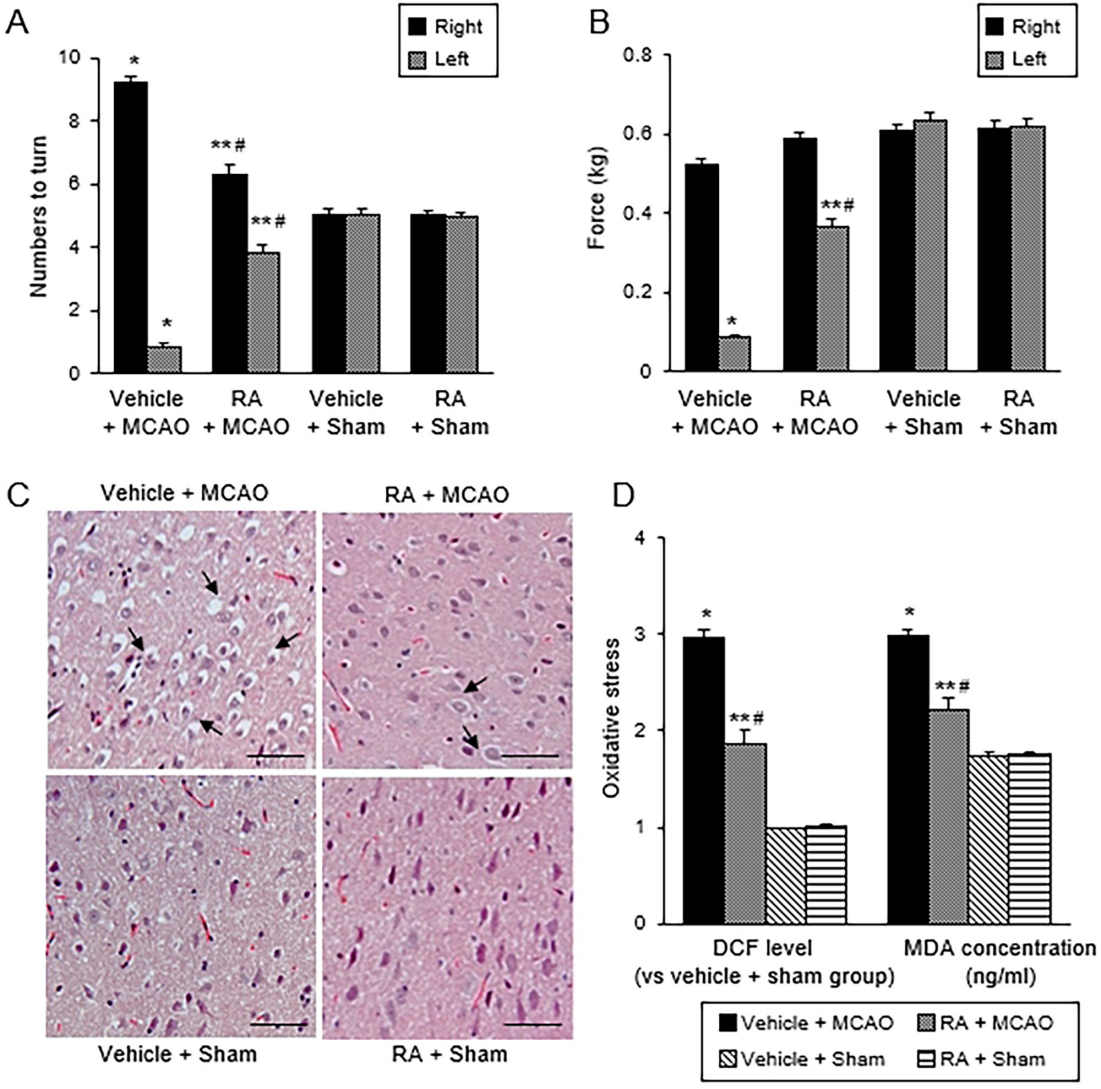
Neurobehavioral tests and oxidative stress analysis. Corner test, grip strength test, hematoxylin and eosin staining, reactive oxygen species (ROS) and lipid peroxidation (LPO) analyses in sham and middle cerebral artery occlusion (MCAO) animals treated with vehicle or retinoic acid (RA). RA alleviated neurobehavioral disorder (A and B) and histopathological changes (C) caused by MCAO damage and attenuated MCAO-induced increases in DCF and MDA levels (D). Arrows indicate damaged cells with vacuoles and swelling. Sham animals have normal neurons with typical morphology. Scale bar = 100 μm. Data (neurobehavioral tests; *n* = 10, oxidative stress analysis; *n* = 5) are presented as means ± S.E.M. *p < 0.01, **p < 0.05 vs. vehicle + sham animals, #*p* < 0.05 vs. vehicle + MCAO animals.

Fig 1 was shown as supplementary data to confirm the neuroprotective effects of retinoic acid in MCAO animals. Cerebral ischemia induces neurobehavioral disorders, including sensorimotor and cognitive dysfunctions. Retinoic acid treatment exerts its neuroprotective effect by preventing MCAO-induced neurobehavioral deficits. Results of the corner test showed that the MCAO animals were characterized by an increased number of right turns due to vibrissae paralysis induced by MCAO. In contrast, retinoic acid treatment prevented vibrissae paralysis and decreased MCAO-induced increase of right turns. There were no significant differences between the sham-operated groups. The number of right turns was 9.19 ± 0.19 in the vehicle + MCAO group and 6.31 ± 0.30 in the retinoic acid + MCAO group (Fig 1A). Retinoic acid also alleviated the MCAO-induced decrease in grip strength of the contralateral forelimb. The force of the left forelimb was 0.08 ± 0.01 kg in the vehicle + MCAO group and 0.36 ± 0.02 kg in the retinoic acid + MCAO group (Fig 1B). However, the force of the right forelimb showed no significant differences in any group. The results of hematoxylin and eosin staining revealed histopathological changes in the right cerebral cortex after right MCAO damage. The right cerebral cortex of the sham-operated group had normal neurons with a pyramidal shape, rounded nuclei, and fully developed dendrites. However, severe histopathological changes, such as vacuolation, cytoplasmic shrinkage, and dendrite deficiencies, were observed in the MCAO group treated with vehicle (Fig 1C). We confirmed that retinoic acid administration alleviated the histopathological changes caused by MCAO damage. We found some damaged cells and normal cells with typical normal morphology in the MCAO group treated with retinoic acid. However, normal cells were also observed in the left cerebral cortex where MCAO surgery was not performed. DCF and MDA contents in the right cerebral cortex were measured to investigate the antioxidant effect of retinoic acid on MCAO damage. MCAO damage increased the levels of DCF and MDA, and retinoic acid treatment attenuated these increases. The level of DCF was 2.97 ± 0.08 in the vehicle + MCAO group and 1.86 ± 0.14 in the retinoic acid + MCAO group (Fig 1D). The MDA level was 2.99 ± 0.06 and 2.21 ± 0.12 in the vehicle + MCAO and retinoic acid + MCAO groups, respectively (Fig 1D).

### Retinoic acid prevents the activation of microglia and astrocytes in the cerebral ischemia

**Fig 2:**
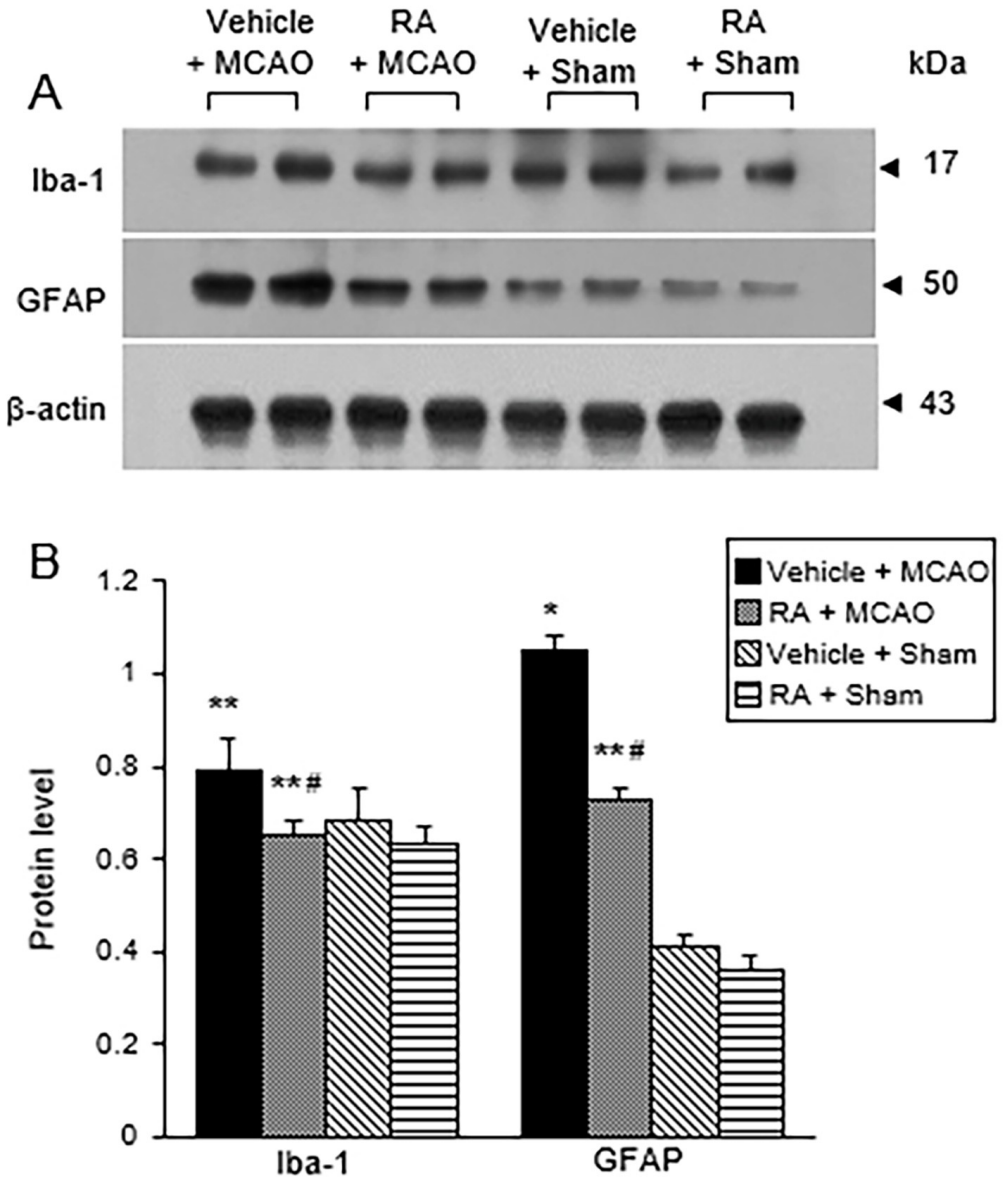
Western blot analysis of glial cell markers in cerebral cortex. Western blot analysis of ionized calcium binding adaptor molecule-1 (Iba-1) and glial fibrillary acidic protein (GFAP) in the right cerebral cortex of sham and middle cerebral artery occlusion (MCAO) animals treated with vehicle or retinoic acid (RA). RA alleviates the increase of Iba-1 and GFAP expression induced by MCAO damage (A). Densitometric analysis results are presented as a ratio of protein intensities to β-actin (B). Data (*n* = 5) are presented as means ± S.E.M. *p < 0.01, **p < 0.05 vs. vehicle + sham animals, #*p* < 0.05 vs. vehicle + MCAO animals.

**Fig 3:**
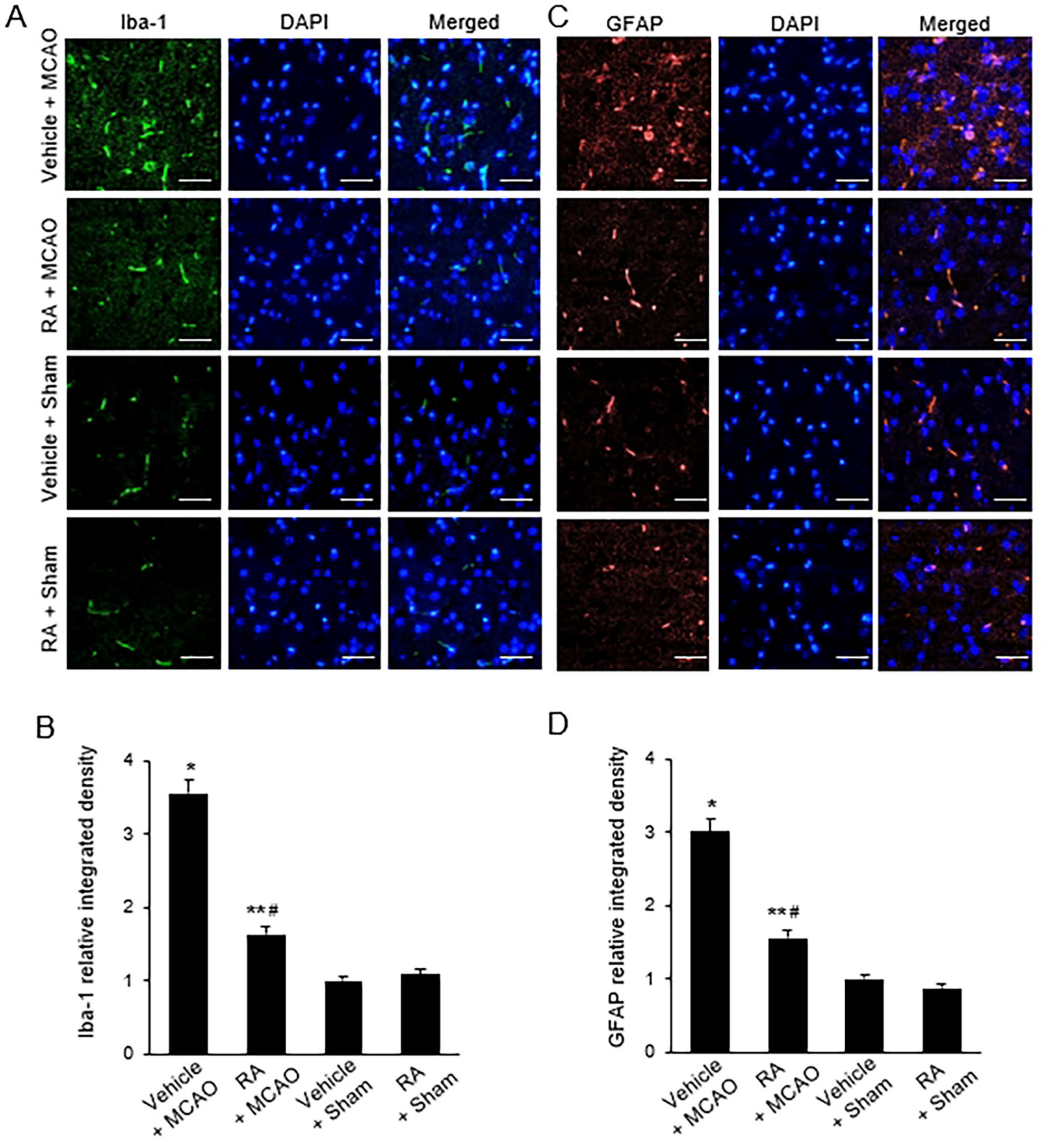
Immunofluorescence of glial cell markers in cerebral cortex. Representative photos of immunofluorescence labeling of ionized calcium binding adaptor molecule-1 (Iba-1) and glial fibrillary acidic protein (GFAP) in the right cerebral cortex of sham and middle cerebral artery occlusion (MCAO) animals treated with vehicle or retinoic acid (RA). RA attenuates the increase of Iba-1 and GFAP expression due to MCAO damage (A and B). The integrated density of Iba-1- or GFAP was expressed as a ratio of the density of each animals to that of vehicle + sham animals (C and D). Scale bar = 100 μm. Data (*n* = 5) are presented as means ± S.E.M. *p < 0.01, **p < 0.05 vs. vehicle + sham animals, #*p* < 0.05 vs. vehicle + MCAO animals.

Western blot analysis and immunofluorescence staining were used to investigate activation of microglia and astrocytes by cerebral ischemia. Western blot analysis results showed that the expression of Iba-1 and GFAP was significantly increased in the MCAO group treated with vehicle (Fig 2A). Retinoic acid treatment significantly ameliorated MCAO-induced increases in Iba-1 and GFAP. The level of Iba-1 was 0.79 ± 0.07 and 0.65 ± 0.03 in the vehicle + MCAO and retinoic acid + MCAO groups, respectively (Fig 2B). The level of GFAP was 1.05 ± 0.03 and 0.73 ± 0.02 in the vehicle + MCAO and retinoic acid + MCAO groups, respectively (Fig 2B). Immunofluorescence staining showed that Iba-1 and GFAP expression was increased in the MCAO group treated with vehicle, and retinoic acid attenuated this increase (Fig 3A and 3B). The integrated density of Iba-1 was 3.56 ± 0.25 and 1.63 ± 0.18 in the vehicle + MCAO and retinoic acid + MCAO groups, respectively (Fig 3C). The integrated density of GFAP was 3.02 ± 0.17 and 1.56 ± 0.12 in the vehicle + MCAO and in the retinoic acid + MCAO groups, respectively (Fig 3D).

### Retinoic acid prevents the activation of pro-inflammatory cytokines in the cerebral ischemia

**Fig 4:**
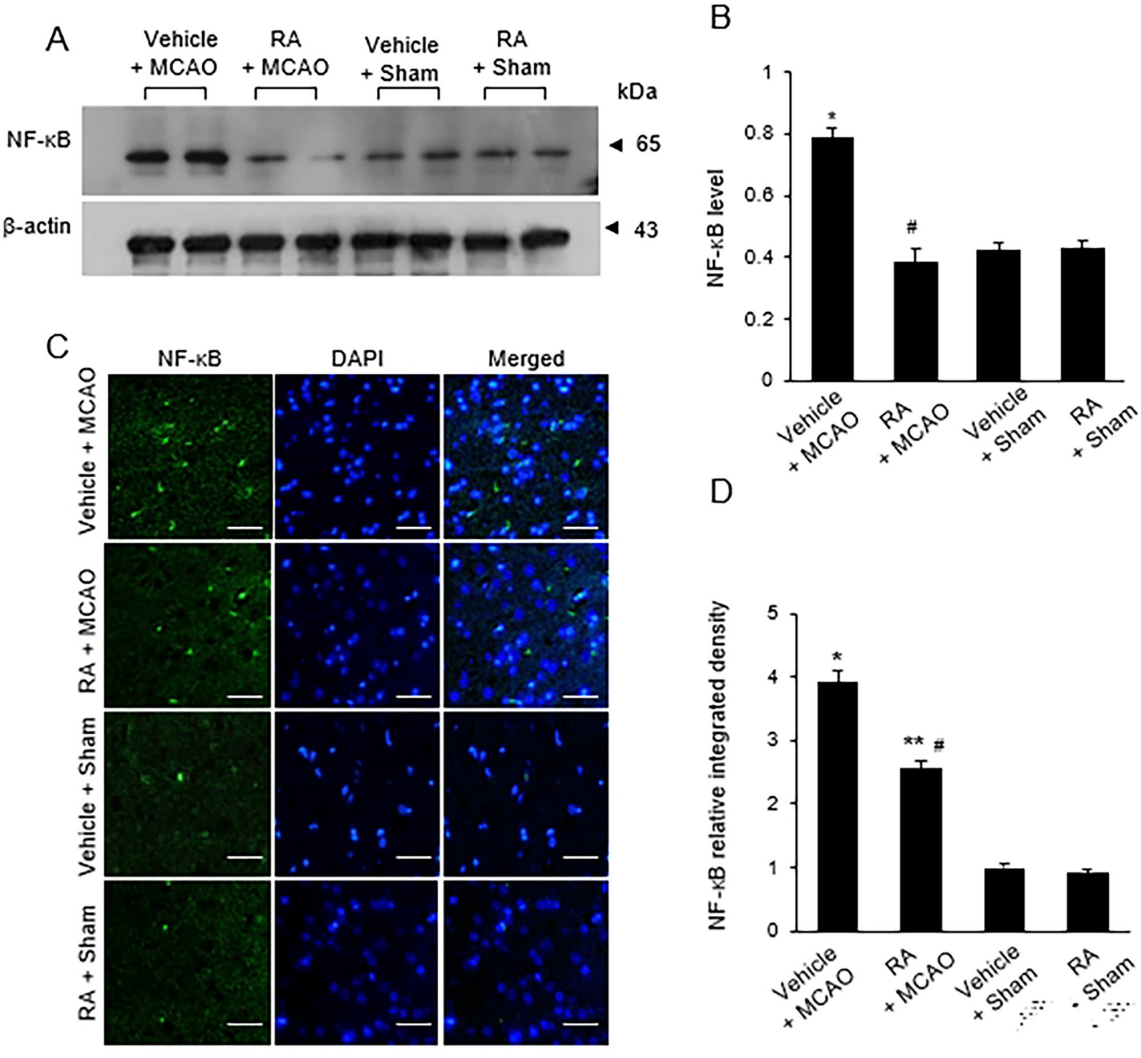
Expression change of NF-κB in cerebral cortex. Western blot analysis and immunofluorescence labeling of nuclear factor kappa B (NF-κB) in the right cerebral cortex of sham and middle cerebral artery occlusion (MCAO) animals treated with vehicle or retinoic acid (RA). MCAO damage increased NF-κB expression, which was alleviated by RA treatment (A and C). Densitometric analysis results are presented as a ratio of protein intensities to β-actin (B). The integrated density of NF-κB was expressed as a ratio of the density of each animals to that of vehicle + sham animals (D). Scale bar = 100 μm. Data (*n* = 5) are presented as means ± S.E.M. *p < 0.01, **p < 0.05 vs. vehicle + sham animals, #*p* < 0.05 vs. vehicle + MCAO animals.

**Fig 5:**
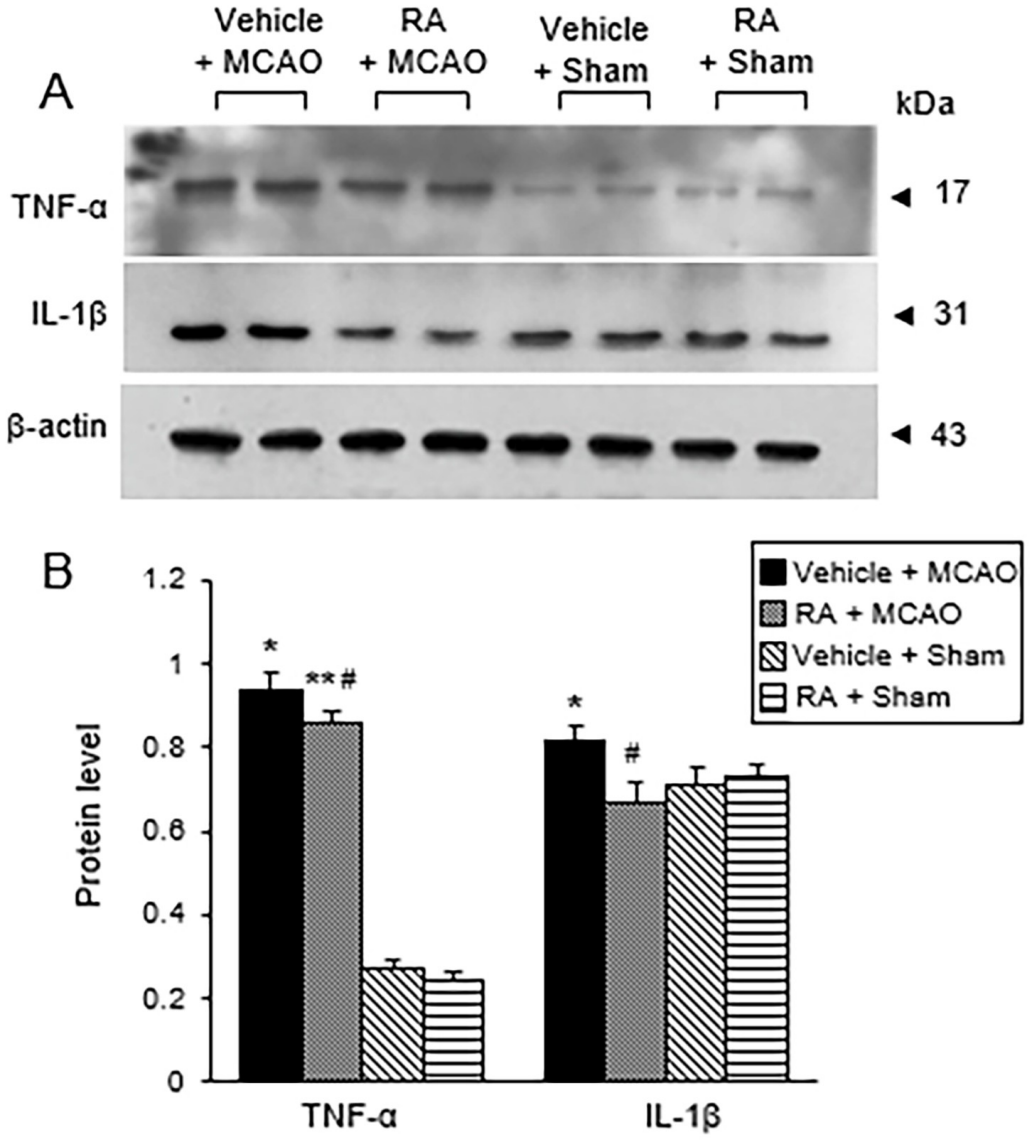
Western blot analysis of TNF-α and IL-1β in cerebral cortex. Western blot analysis of tumor necrosis factor-α (TNF-α) and interleukin-1β (IL-1β) in the right cerebral cortex of sham and middle cerebral artery occlusion (MCAO) animals treated with vehicle or retinoic acid (RA). RA alleviates the increase of IL-1β and TNF-α expression induced by MCAO damage (A). Densitometric analysis results are presented as a ratio of protein intensities to β-actin (B). Data (*n* = 5) are presented as means ± S.E.M. *p < 0.01, **p < 0.05 vs. vehicle + sham animals, #*p* < 0.05 vs. vehicle + MCAO animals.

**Fig 6:**
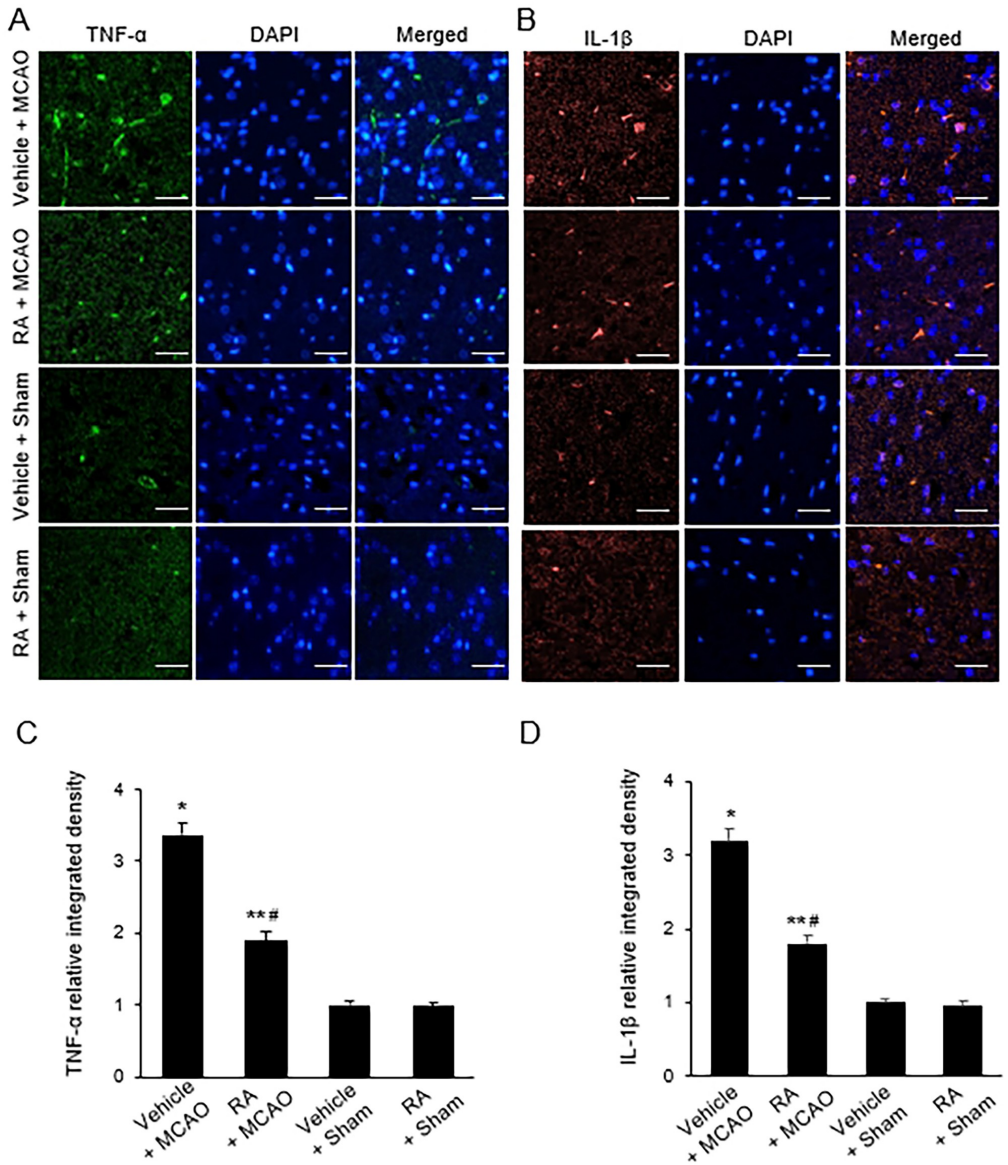
Immunofluorescence of TNF-α and IL-1β in cerebral cortex. Representative photos of immunofluorescence labeling of tumor necrosis factor-α (TNF-α) and interleukin-1β (IL-1β) in the right cerebral cortex of sham and middle cerebral artery occlusion (MCAO) animals treated with vehicle or retinoic acid (RA). RA attenuates the increase of TNF-α and IL-1β expression due to MCAO damage (A and B). The integrated density of TNF-α or IL-1β was expressed as a ratio of the density of each animals to that of vehicle + sham animals (C and D). Scale bar = 100 μm. Data (*n* = 5) are presented as means ± S.E.M. *p < 0.01, **p < 0.05 vs. vehicle + sham animals, #*p* < 0.05 vs. vehicle + MCAO animals.

MCAO also induced a significant increase in NF-κB, while retinoic acid attenuated this increase (Fig 4A). Western blot analysis revealed levels of NF-κB in the vehicle + MCAO and in the retinoic acid + MCAO groups of 0.78 ± 0.05 and 0.38 ± 0.07, respectively (Fig 4B). Immunofluorescence staining results were similar to those of Western blot (Fig 4C). The integrated density of NF-κB-positive cells was 3.93 ± 0.18 in the vehicle + MCAO group and 2.56 ± 0.15 in the retinoic acid + MCAO group (Fig 4D). Figs 5 and 6 show the changes of TNF-α and IL-1β increases by MCAO damage in the presence of retinoic acid. MCAO damage increased the expression of these proteins, while retinoic acid treatment alleviated these increases. The expression levels of TNF-α and IL-1β were determined by Western blot analysis (Fig 5A). The level of TNF-α was 0.94 ± 0.04 in the vehicle + MCAO animals and 0.87 ± 0.03 in the retinoic acid + MCAO animals (Fig 5B). The level of IL-1β was 0.82 ± 0.03 in the vehicle + MCAO animals and 0.67 ± 0.05 in the retinoic acid + MCAO animals (Fig 5B). The results of immunofluorescence staining showed increases in TNF-α and IL-1β caused by MCAO damage. These increases were attenuated by retinoic acid treatment (Fig 6A and 6C). The integrated density of TNF-α was 3.37 ± 0.06 and 1.89 ± 0.15 in the vehicle + MCAO and retinoic acid + MCAO animals, respectively (Fig 6B). The integrated density of IL-1β was 3.18 ± 0.19 in the vehicle + MCAO animals and 1.80 ± 0.14 in the retinoic acid + MCAO animals (Fig 6D).

## Discussion

We have previously reported the protective effect of retinoic acid on cerebral ischemia [24]. Fig 1 was provided as a basic data for confirming the neuroprotective effect of retinoic acid in cerebral ischemia. Serious behavioral disorders were observed during ischemic stroke, and these neurobehavioral disorders were markedly improved in the presence of retinoic acid. Furthermore, cerebral ischemia caused significant histopathological changes in neurons in the cerebral cortex, while retinoic acid treatment protected cortical neurons against these damages. Ischemic injury induces oxidative stress, causes mitochondrial dysfunction, and accelerates the inflammatory response [31, 32]. Retinoic acid exerts a powerful antioxidant effect on various harmful substances, such as hydrogen peroxide and staurosporine in nerve cells [17, 33, 34]. We confirmed that retinoic acid prevented MCAO-induced increases in DCF and MDA levels. Thus, our findings showed that retinoic acid alleviated the oxidative stress caused by ischemic injury. Furthermore, a previous study has shown that administration of retinoic acid 24 h after MCAO surgery has a neuroprotective effect by regulating LINGO-1 protein and inducing neural regeneration [35]. Retinoic acid administration before and after MCAO reduces MCAO-induced cerebral infarction [36, 37]. These studies have shown that the administration of retinoic acid after MCAO surgery has neuroprotective effects. In this study, we focused on the preventive effect of retinoic acid in stroke animal models and evaluated the effect of retinoic acid by administering retinoic acid before MCAO surgery.

Ischemic stroke increased the activity of microglia and astrocytes [38, 39]. Activation of microglia and astrocytes causes an inflammatory response and nerve cell damage [40, 41]. Overactivation of microglia by ischemic stroke induces excessive phagocytosis, increases blood-brain barrier permeability, and increases cytokine and oxidative metabolite secretions [38]. We found an increase in the expression of Iba-1 and GFAP in the presence of MCAO damage. These proteins are common markers of activated microglia and astrocytes, respectively [40, 41]. Increased expression of Iba-1 and GFAP by ischemic injury induces proliferation of microglia and astrocytes, stimulates the expression of NF-κB, and releases inflammatory cytokines including TNF-α and IL-1β [42]. Inhibiting the hyperactivation of microglia and astrocytes reduces brain damage caused by ischemic injury [43]. Retinoic acid attenuated the MCAO-induced increase in Iba-1 and GFAP expression. Our results suggest that retinoic acid inactivates microglia and astrocytes by attenuating Iba-1 and GFAP expressions. Retinoic acid inhibits the expression of Iba-1 and GFAP by interacting with its receptor α, regulates autophagy activation and the JAK-STAT signaling pathway, and protects neuronal cells against damage [44, 45]. These studies clearly supported our finding that retinoic acid modulated the expression of Iba-1 and GFAP in ischemic conditions.

Inflammation is involved in neurodegenerative diseases, such as Alzheimer’s disease and ischemic stroke [7, 46]. NF-kB is a transcription factor that is highly expressed in all mammalian cells. It is a major regulator of various genes involved in immune and inflammatory responses [47]. NF-kB regulates the expression of genes related to the cell cycle and apoptosis and is a major mediator of pro-inflammatory cytokines such as TNF-α, IL-1β, IL-6, and IL-8 [48]. Increased NF-κB level induces inflammatory responses and causes autoimmune diseases and cancer [49]. In addition, NF-κB activation affects glial cells activation and induces neuronal cell death in focal cerebral ischemia [50, 51]. We confirmed that MCAO damage increases NF-κB expression, and retinoic acid attenuates this increase. These results suggest that retinoic acid modulates the expression of NF-κB and alleviates the inflammatory response.

TNF-α and IL-1β are pro-inflammatory cytokines released by NF-κB activation. Ischemic stroke increases the release of TNF-α and IL-1β, which influence the severity of neurological deficits and cell damage through the production of free radicals [52, 53]. Regulation of TNF-α and IL-1β expression also affects neuroprotection in ischemic brain injury. The reduction of TNF-α protects against blood-brain barrier disruption, decreases apoptosis protein activation, and decreases neuronal cell death [54]. Overexpression of IL-1β increases the incidence of cerebral infarction and induces pro-inflammatory mediators including leukotrienes and platelet activation factors, while blockage of IL-1β attenuates ischemic brain injury and alleviates neutrophil infiltration in MCAO injury [55]. We found an increase in TNF-α in MCAO damage, and retinoic acid alleviated this increase. In addition, retinoic acid inhibits the increase of IL-1β caused by MCAO damage. Retinoic acid also prevents inflammation by inhibiting TNF-α and IL-1β in osteoarthritis models [56]. Retinoic acid attenuates neuroinflammation by inhibiting the increase of inflammatory cytokines such as TNF-α and IL-6 induced by cerebral ischemic injury [22]. We also identified significant increases in NF-κB, TNF-α, and IL-1β in cerebral ischemia, and retinoic acid significantly alleviated these increases. These results were confirmed by Western blot and immunofluorescence staining and suggest that retinoic acid prevents neuroinflammation in cerebral ischemic injury by regulating the expression of NF-κB, TNF-α, and IL-1β. Taken together, these results demonstrate that retinoic acid reduces oxidative stress and inhibits the activation of microglia and astrocytes in the presence of MCAO damage. Retinoic acid also prevents increases of NF-κB and pro-inflammatory cytokines TNF-α and IL-1β.

## Conclusion

Ischemic stroke induces the activation of glial cells and increases pro-inflammatory cytokines, while retinoic acid alleviates these changes. Therefore, our findings suggest that retinoic acid exerts antioxidant and anti-inflammatory effects by modulating the activity of glial cells and inflammatory cytokines in an ischemic stroke model.
